# Supplementary material for: Epidemiology, Clinical, and Microbiological Characteristics of Multidrug-Resistant Gram-Negative Bacteremia in Qatar
Source: Antibiotics (Basel). 2024 Mar 31;13(4):320. doi: 10.3390/antibiotics13040320 (PMC11047403; doi:10.3390/antibiotics13040320)
Supplement: Supplementary file 1 [file antibiotics-13-00320-s001.zip › antibiotics-2915516-supplementary.pdf]

Figure S1: Study flow chart of MDR Gram negative bacteria blood stream infections collected during 2019.

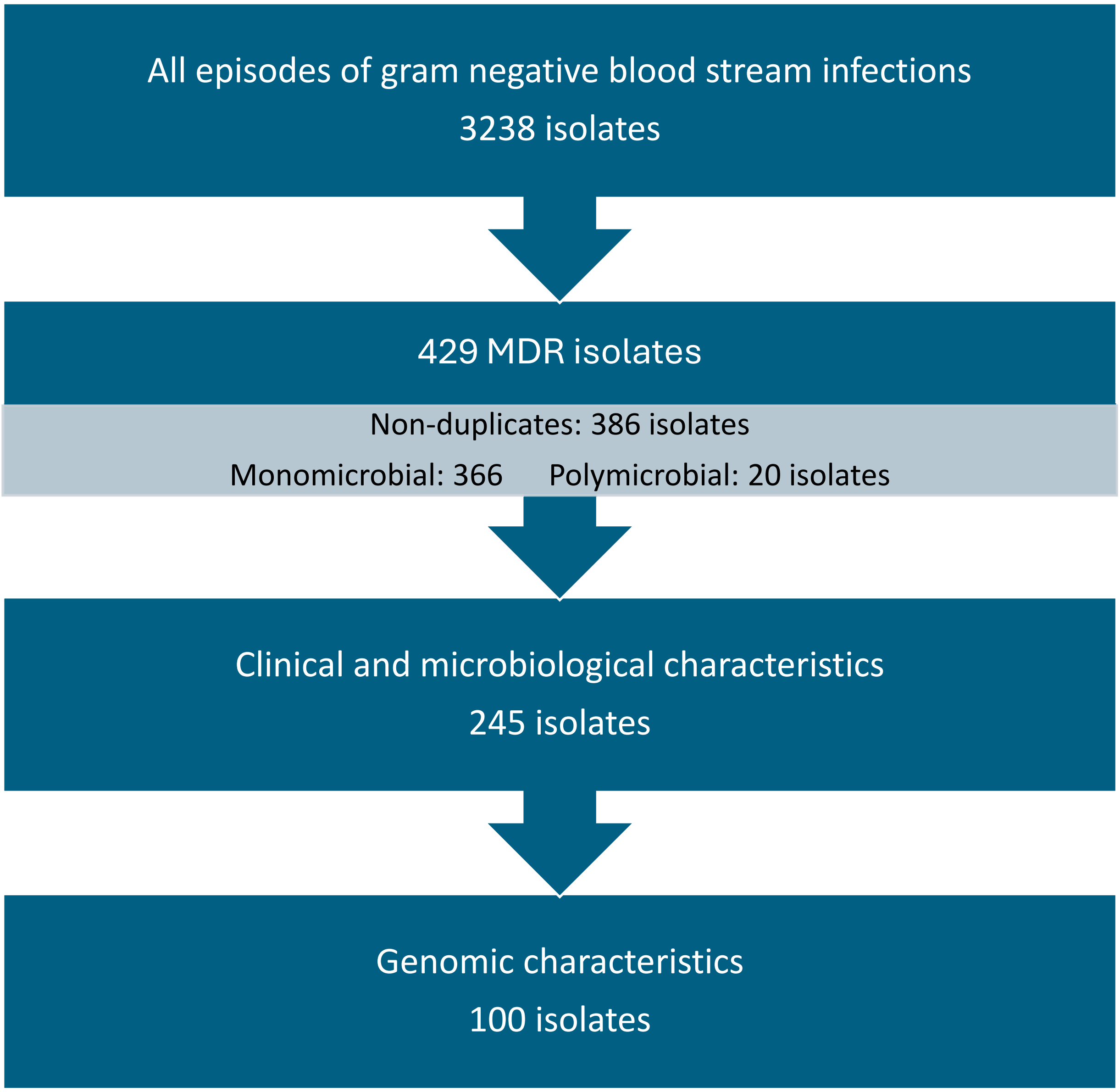

MDR : multidrug resistant
